# Triggering avalanches by transverse perturbations in a rotating drum

**DOI:** 10.1038/s41598-021-93422-2

**Published:** 2021-07-06

**Authors:** Vicente Salinas, Cristóbal Quiñinao, Sebastián González, Gustavo Castillo

**Affiliations:** 1 Instituto de Ciencias Químicas Aplicadas, Facultad de Ingeniería, Universidad Autónoma de Chile, Santiago, Chile; 2 Instituto de Ciencias de la Ingeniería, Universidad de O’Higgins, Rancagua, Chile; 3 Dipartimento di Elettronica Informazione e Bioingegneria, Politecnico di Milano, Milan, Italy

**Keywords:** Fluid dynamics, Statistical physics, thermodynamics and nonlinear dynamics

## Abstract

We study the role of small-scale perturbations in the onset of avalanches in a rotating drum in the stick-slip regime. By vibrating the system along the axis of rotation with an amplitude orders of magnitude smaller than the particles’ diameter, we found that the order parameter that properly describes the system is the kinetic energy. We also show that, for high enough frequencies, the onset of the avalanche is determined by the amplitude of the oscillation, contrary to previous studies that showed that either acceleration or velocity was the governing parameter. Finally, we present a theoretical model that explains the transition between the continuous and discrete avalanche regimes as a supercritical Hopf bifurcation.

Dry granular systems are usually defined as a collection of macroscopic particles that interact mainly through dissipative collisions. Despite its simplicity, they possess a wide variety of behaviors. Depending on the dissipation and injection of energy, they may behave as solids, liquids, and gases^1,2^. One example where this is observed is the case of a rotating drum. It is quite known that in the absence of external vibrations and depending on the rotation speed, the system presents a variety of regimes^3^. At very low rotation speeds, the system displays what is called a stick-slip or slumping behavior, where the slope of the free surface fluctuates periodically between two angles, the angle of marginal stability and the repose angle^4–6^. During the build-up, almost all the grains move in a rigid-solid-like way with the drum, to suddenly release the energy in the form of an avalanche. By increasing the rotation speed, the system undergoes a transition to a regime where there is a continuous flow of grains (rolling), that displays a flat free surface and whose slope depends on the angular velocity^4,7^. In this regime, only a small number of grains is involved in the flow, defining a fluid-like and a solid-like zone^2^. The effect of mechanical vibrations, in a suitable range of amplitudes and frequencies, has been proven to increase the mobility of grains, reducing the contact area, and thus reducing considerably the friction^8,9^. In the present work we show the dependence between the critical rotational velocity required to transit between the two states in a rotating drum experiment as a function of small transverse perturbations. The order parameter governing such a transition is discussed and an analytical model for such behavior is proposed based on a well known Sel’kov model.

## Results

### Discrete element simulations

To study the problem of granular avalanches, discrete element method simulations (DEM)^10^ were performed. In this method, the translational and angular movement of each particle are described by Newton’s equation and Euler’s equations, respectively. Contacts are modelled by means of a soft-sphere approach so that the particles can be slightly overlapped. The DEM code used in this work is the open-source software MercuryDPM^11–13^. The contact model used is the standard linear spring dash-pot model^11,14,15^.

**Figure 1:**
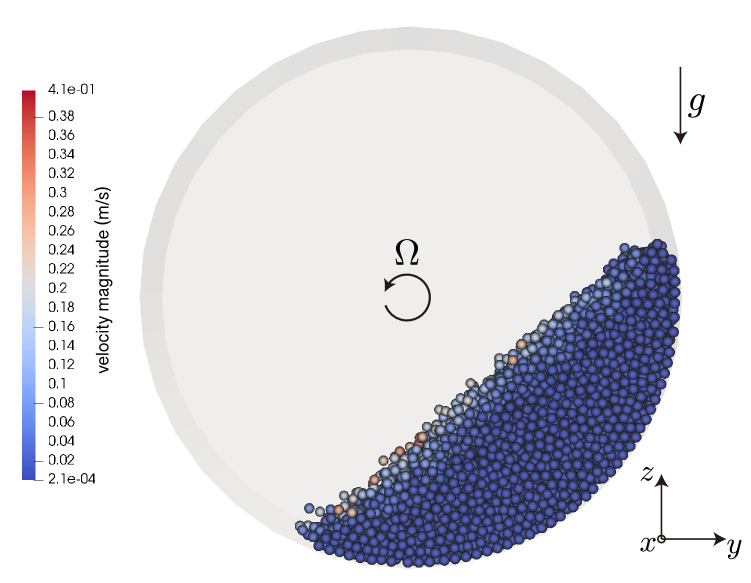
Snapshot of the rotating drum obtained with MercuryDPM. The imposed transverse forcing is along the $x-$direction, such that the movement of the walls is $x(t) = A\sin (2\pi f t)$. In this case, $\Omega = {1}\hbox { rpm}$ and $A=0$.

We consider a drum filled with $N=4500$ spherical particles of $d={1}\hbox { mm}$. The drum has a diameter $R=50 d$ and length $h=10d$, thus giving a filling fraction of $12\%$. The direction of the vibration is perpendicular to the gravity and parallel to the rotation axis. The system is forced sinusoidally with displacement $x(t) = A\sin ( 2\pi f t)$ (see Fig. 1). The forcing frequency is either $f = {120}\hbox { Hz}$ or $f = {240}\hbox { Hz}$, while the amplitude *A* explored ranges from ${0.0}\,{\upmu }\hbox {m}$ to ${1.7}\,{\upmu }\hbox {m}$, far smaller than a particle’s diameter. Thus, the dimensionless acceleration $\Gamma = A(2\pi f)^2/g$ ranges from 0 to $9.6\times 10^{-2}$. The total simulation time was set to depend on the rotation speed so that for each simulation run the drum completes two whole turns. All the walls are solid (non periodic), smooth and frictional. The wall’s imposed frequency movement is obviously limited by the time-step, but we are well below this limit ($1/t_c = {10^5}\hbox { Hz}$). The normal and tangential restitution coefficients are the same and set to $e = 0.7$. The parameters used in the simulations are specified in Table 1.

**Table 1:** Parameters used in DEM simulations.

Density	${2500}{\hbox { kg}/\hbox {m}^{3}}$
Coefficient of restitution	0.7
Sliding friction particle–particle	0.74
Sliding friction particle–wall	0.40
Rolling friction particle–particle	0.15
Rolling friction particle–wall	0.01
Collisional time	$5\times 10^{-4}\, \hbox { s}$
Time-step	$1\times 10^{-5}\, \hbox { s}$

### Slipping to rolling transition

**Figure 2:**
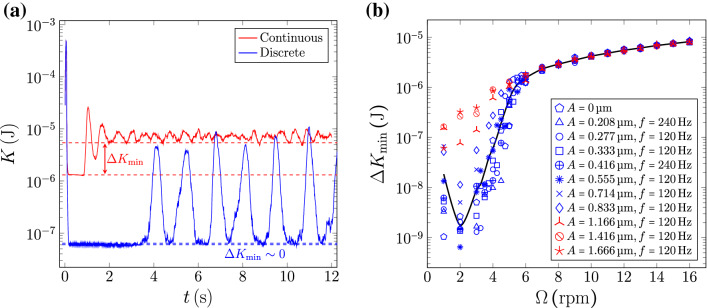
(**a**) Total kinetic energy of the system for both discrete and continuous cases. The discrete regime corresponds to $A = {0.8}\,{\upmu }\hbox {m}$ and $\Omega = {2}\hbox { rpm}$, whereas the continuous regime corresponds to $A = {0.8}\,{\upmu }\hbox {m}$ and $\Omega = {10}\hbox { rpm}$. (**b**) $\Delta K_{\mathrm {min}}$ for different forcing amplitudes and frequencies. It is observed that, depending on the forcing amplitude, there are two distinguishable data sets. $\Delta K_{\mathrm {min}}$ is very similar for low amplitudes, whereas it jumps for amplitudes larger than $A\approx {1}\,{\upmu }\hbox {m}$. The increment of $\Delta K_{\text {min}}$ at the smallest value of $\Omega$ is just apparent; it is the energy of just one particle falling a distance of one diameter. The continuous black curve corresponds to a smoothing spline of all the collapsing data (blue data).

To study the effect of mechanical vibrations and the grain mobility near the transition, we applied a transverse perturbation to the system. We observe that for a given perturbation amplitude, there is a critical rotation speed below which the system presents discrete avalanches. Otherwise, the system displays a continuous regime. To characterize this transition, we measure the total kinetic energy of the system, defined by $K = \sum _i K_i = \sum (1/2)m_iv_i^2$ where $m_i$ and $v_i$ represent the mass and speed of each particle respectively. The kinetic energy is presented in Fig. 2a for both discrete and continuous cases.

We observe that, once the system has reached a steady state, for the slumping regime, the potential energy displays a sawtooth behavior related to the start and end of the avalanches. In contrast, for the continuous regime, *K* reaches a constant value, related to the position of the centre of mass of the system. Something similar is observed in the total gravitational energy, see Fig. 4a and Supplementary Material. We observe that, once the system has reached a steady state, for the discrete avalanche regime, the kinetic energy displays huge peaks, while for the continuous regime, *K* presents much smaller oscillations. The rapid increase and decrease of the kinetic energy correspond to an avalanche event (sudden change in the bed slope), while the time where *K* remains almost constant corresponds to the build-up process (gradual change in the bed slope). By defining $\Delta K_{\mathrm {min}}$ as the difference between the base energy and the minima of the kinetic energy when the system has reached the steady state (see Fig.  2a), we study how the system transits from the discrete to the continuous regime. In Fig. 2b we show the behavior of $\Delta K_{\mathrm {min}}$ as a function of $\Omega$ for different forcing parameters. It is observed that, depending on the forcing, there are two clearly distinguishable behaviors. As the forcing amplitude is increased, at some value $A\sim {1}\,{\upmu }\hbox {m}$, $\Delta K_{\mathrm {min}}$ changes its behavior and jumps to much larger values. It is worth noting that for very large forcing amplitudes, for the rotation speeds explored, we observe only the continuous regime.

We propose that the control parameter for transitioning from stick-slip to continuous flow is the forcing amplitude. To support this statement, we will analyze Fig. 2b in detail: (1) It is possible to verify that for measurements with amplitudes smaller than ${1}\,{\upmu }\hbox {m}$, the same behavior is observed when the system is forced at a different velocity or acceleration. For example, as shown by Table 2, the dataset e) has twice the speed of b), however they show the same behavior. (2) the dataset b) has the same acceleration as h), but their behaviors are different. (3) e) has the same velocity as h), but they show different behavior. This analysis allows us to rule out velocity and/or acceleration as the relevant parameter to control the regime change in the system, leaving the forcing amplitude as the control parameter.

**Table 2:**
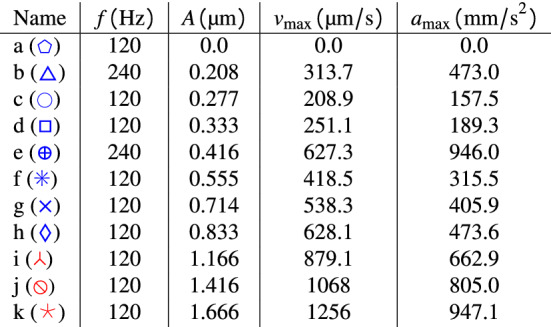
Forcing parameters imposed on the system.

The maximum speed and acceleration imposed on the drum are $v_{\text {max}}=(2\pi f)A$, and $a_{\text {max}}=(2\pi f)^2A$ respectively. Note that the data {a, b, c, d, e, f, g, h} have blue markers in Fig. 2b, whereas the data {i, j, k} have red markers.

As a probable explanation for this behavior, we propose that the fact that this transition occurs at such small amplitudes is related to the typical size of the asperities, the roughness of the grains. Once the amplitude of the forcing is larger than the asperity size, the grains are no longer locked up, the force chains break up, and the system transits to the continuous regime. Notice that, even though there is no explicit rugosity in the simulations, friction plays an analogous role. According to^16^, for particles with diameter $d\simeq {1}\hbox { mm}$, the rugosity is of the order $\simeq {1}\,{\upmu }\hbox {m}$, which is consistent with our interpretation. For our simulations, the number of contacts with an overlap smaller than ${1}\,{\upmu }\hbox {m}$ is around the $27\%$ of the contacts in the system (see Supplementary Material). This means that a significant fraction of the particles can be “freed” of their contacts when exciting at this amplitude. In the real world, this scale is associated to the rugosity of the particles and therefore the viscoelastic model (linear or Hertz) is not applicable since the particles have not had the chance to deform yet; only their surface roughness is in contact. The veracity of this claim remains to be experimentally validated yet seems plausible.

On the other hand, the timescale of this vibration is also relevant for a successful breaking up of the force chains. The typical timescale in a granular system may be estimated as the time it takes for one grain to fall over another grain due to gravity, which in our case is $\tau _g=\sqrt{2d/g} = {14.3}\hbox { ms}$. This corresponds to a frequency of $f_g = 1/\tau _g = {70}\hbox { Hz}$. When applied to rocks of $\sim {10}\hbox { cm}$, the associated frequency is ${7}\hbox { Hz}$. Thus, in order to successfully break up the force chains and prevent the system from rearranging itself, the forcing should be done at frequencies larger than $f_g$. Therefore, we can conclude that as long as the forcing frequency is larger than $f_g$, the governing parameter in the slumping-rolling transition at a fixed rotation speed is the imposed amplitude.

**Figure 3:**
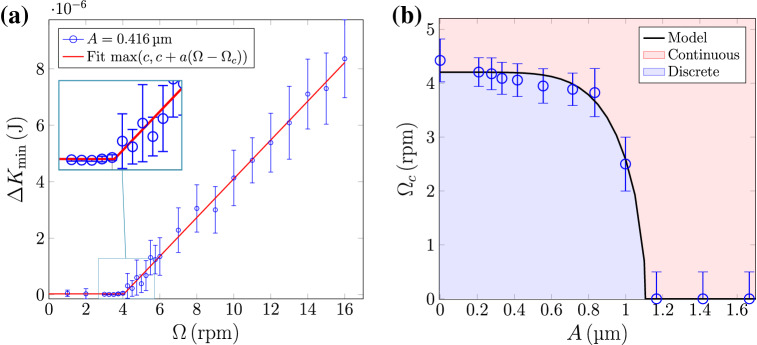
(**a**) Minimum of the total kinetic energy for $A = {0.416}\,{\upmu }\hbox {m}$. For the sake of clarity in the figure, the other amplitudes are not plotted. By fitting the linear piecewise function $\Delta K_{\mathrm {min}} = \max {(c,c+a(\Omega -\Omega _c))}$ it is possible to obtain the critical rotation speed, $\Omega _c$, for each amplitude where the transition is observed ($A\lesssim {1}\,{\upmu }\hbox {m}$). (**b**) Critical rotation speed, $\Omega _c$, as a function of the forcing amplitude $A$. The continuous black line corresponds to the theoretical model described in the following section.

Additionally, by taking a closer look at what happens at rotation speeds near the transition, we get a very distinctive change of behavior in $\Delta K_{\mathrm {min}}$. From Fig. 3a we can see that, for amplitudes where the transition is observed ($A\lesssim {1}\,{\upmu }\hbox {m}$), $\Delta K_{\mathrm {min}}$ presents two clear distinguishable linear behaviors. For each forcing, $\Delta K_{\mathrm {min}}$ displays a slow linear trend for low rotation speeds, while at higher rotation there is a break in the slope. By fitting a piecewise linear function (the equation $\Delta K_{\mathrm {min}} = \max {(c,c+a(\Omega -\Omega _c))}$, where $\max$ represents the maximum function), we can obtain the critical rotation speed $\Omega _c$. This is shown in Fig.  3b. We can see that the critical speed, $\Omega _c$, above which the system displays continuous avalanches, decreases as the forcing is increased. Thus, as the forcing is increased, it becomes easier to reach the rolling regime. In other words, less energy coming from the rotation is required to reach the transition. Moreover, it is also observed that there is a maximum value of *A* above which it is impossible to observe discrete avalanches, regardless of how small the rotation is. There is so much energy injected into the system by means of the imposed forcing, constantly breaking up the chain forces that might form, that the system is always in the continuous regime.

When the system is in the stick-slip regime, it shares some key elements with self-oscillation dynamics. Indeed, at the start of an avalanche, the kinetic energy increases due to the falling particles. The larger the number of falling particles is, the more it grows. Eventually, it becomes so large that no more particles can fall. In a self-oscillator system the faster the object moves, the more it is pushed along the direction of its motion. The oscillation amplitude grows exponentially with time until it becomes so large that nonlinear effects become relevant, resulting in a self-regulated periodic motion. The framework has been successfully used to describe, among other phenomena, the human voice and clocks, musical instruments, the heart, motors, and the theory of lasers^17^. Using this analogy, by modifying the classical Sel’kov model^18^ (which itself is an extension of the well known Lotka-Volterra model) we find that the critical rotational speed can be characterised by a Hopf transition; from a continuous avalanche for large amplitudes, to a two-regime region for low enough amplitudes.

### Self-oscillation theoretical analogy

We present a simple model that captures the essence of the two regimes and the transitions between them as discussed previously. Starting with the well-known model of Sel’kov^18^ to describe self-oscillations in Glycolysis, we propose that the most prominent properties of the energy dynamics (see Fig. 2) can be described qualitatively by a two dimensional, slow-fast system of ordinary differential equations. In order to study the unperturbed rotating drum, consider the following model:

$$\begin{aligned} \dot{u}&= \epsilon \big (g(\Omega )-(1+v^2)u\big ), \\ \dot{v}&= -v+(1+v^2)u~. \end{aligned}$$

The variable *u* has to be understood as a functional representation of the gravitational potential energy of the system, while *v* is representing the total kinetic energy. The function $g(\Omega )$ is related to the supply of gravitational potential energy due to the drum rotation. From a kinetic perspective, $g(\Omega )$ acts as a source term to the *u* variable that increases with the angular velocities $\Omega$. The constant $\epsilon$ acts as a slow-fast dimensionless quantity. We observe that the time-scale of avalanches is much faster than the change in potential energy from experiments. By taking $\epsilon$ small and fixed, we force the system to allow slow variations on *u* by maintaining *v* relatively constant on the respective manifold. However, the bifurcation results here exposed remain true for $\epsilon$ close to zero.

Qualitatively, the dynamical system behaves as follows: In the first stage, *u* increases almost linearly at a rate $\epsilon g(\Omega )$. At some point, the term $(1+v^2)u$ dominates, and *v* increases rapidly. As *v* increases, the right-hand side of the first equation becomes negative, and a sudden decrease in *u* occurs. Finally, the second equation comes back to equilibrium, and the cycle starts again. In order to obtain the sawtooth behavior of the variable related to the potential energy, we have used the notion of self-excitation and cross-inhibition dynamics. There are two effects related to the term $v^2u$: as the drum rotates, some of the particles start to fall, thus decreasing the gravitational potential energy and, at the same time, increasing the total kinetic energy of the system. As the kinetic energy increases, more and more particles start to move, showing a well-known self-excitation phenomenon^19^. However, this effect decreases the total potential energy until no more particles can fall, i.e. the slope of the system becomes flat.

**Figure 4:**
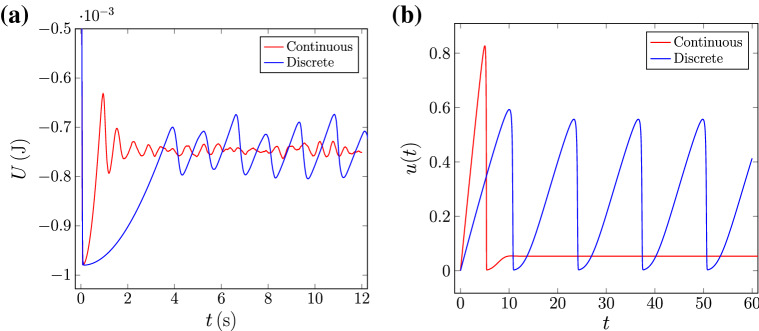
(**a**) Potential energy of the system for both continuous and discrete regimes. The discrete regime corresponds to $A= {0.8}\,{\upmu }\hbox {m}$ and $\Omega = {2}\hbox { rpm}$, whereas the continuous regime corresponds to $A = {0.8}\,{\upmu }\hbox {m}$ and $\Omega = {10}\hbox { rpm}$. (**b**) Numerical solution of *u*(*t*) to the proposed theoretical model for $\epsilon =0.01$, $A=0.8$ and $\Omega =2$ and 10.

Now we discuss how to modify the original system to incorporate horizontal vibrations. As the drum rotates, we have stated that the energy is injected mainly as a gravitational potential source term that later is transformed by an internal dynamical process into kinetic energy. Therefore, if we perturb the system through vibrations, we have to incorporate a new source term into the mathematical description. The general form to add this new configuration is to plug a source term on the form *f*(*A*) on the second equation. To summarise, we propose to change the unperturbed model as follows:

$$\begin{aligned} \dot{u}&= \epsilon \big (g(\Omega )-(1+v^2)u\big ),\\ \dot{v}&= f(A)-v+(1+v^2)u. \end{aligned}$$

The chosen term captures the most prominent aspect of the system (steady states depending on *A* and $\Omega$), and it is still simple enough to have tractable analytical conditions to (1) the apparition of a limit cycle and (2) bifurcations/stability conditions. To go further in the understanding of the apparition of a limit cycle, we solve the system by adapting some classical techniques for two-dimensional systems (see e.g., Strogatz, S.^20^).

The transition between the continuous and discrete regimes defines a curve in the $(f(A), g(\Omega ))$ space. Using classical bifurcation analysis, we see that the transition happens in the two following cases: either through large enough vibrations *A* or fast enough rotating speeds $\Omega$. We propose the following functions $f(A) = A^k,\, g(\Omega ) = r_1(\Omega +r_2)$. By fixing $\epsilon = 0.01$, the parameters that best fit the phase diagram from Fig. 3b are $r_1=1.4109$, $r_2=2.7767$, and $k=5.6663$. As it can be seen, there is a good qualitative agreement between the simulation data and the proposed analytical model. For $\Omega < \Omega _c$ the regime of discrete avalanches is present, whereas for $\Omega > \Omega _c$ the limit cycle looses its stability and the continuous regime emerges. It is important to remark here that $\epsilon$ controls the rate at which *u* changes. Any choice of $\epsilon$ smaller than 0.125 displays the same qualitative behavior (See Supplementary Material). Please note that this model is not the only one that fits the data set. However, it additionally fits the bistability behavior of the kinetic and potential energies, characteristic of the stick-slip system.

Figure 4 shows a comparison between the DEM simulations and the numerical solution to the theoretical model for two different values of $\Omega$ at fixed $A=0.8$. The numerical solutions are obtained through a Runge–Kutta 4 scheme for $\Omega =2,$ and $\Omega =10$. We obtain a similar qualitatively behavior in terms of the two states, discrete and continuous, albeit the scales of the variables are different. Furthermore, the discrete state has the same sawtooth structure of the potential energy. Finally, notice that in the experiment the continuous state is noisy whereas our model is lacking noise in the equations and thus remains constant.

## Conclusions

We have presented evidence that the stability of a pile of grains, hence the macroscopic friction of the system, might be altered by means of tiny transverse vibrations. By using the minimum of the kinetic energy of the system as a key parameter to describe the transition, we found that for very low perturbations $(\lesssim {1}\,{\upmu }\hbox {m})$, the system transits from a slumping to a rolling regime. This highlight the importance of how local rearrangements affect the macroscopic response of granular media. In other words, through small perturbations and without fluidizing the system, we could modify the avalanche angles^6^ and hence the effective friction coefficients in the system. From the simulations, by using a quite clean method, we could also obtain the critical rotation speed $\Omega _c$. We also found that the governing parameter behind the transition is the forcing amplitude, as opposed to previous research has stated that the quantity that controls the frictional properties of similar granular systems are either the imposed acceleration^21^ or the imposed velocity^22^.

Additionally, we presented a model that captures the essential physics behind the observed regimes. It describes the transition as a Hopf bifurcation, and with it, we get oscillatory/continuous behaviors in good correspondence with what we get from the simulations. These results could shed some light on the understanding of how and under what circumstances earthquakes destabilize sandpiles on hills to produce landslides^23^.

## Supplementary Information

Supplementary Information 1.
