# Supplementary material for: Triggering avalanches by transverse perturbations in a rotating drum
Source: Sci Rep. 2021 Jul 6;11:13936. doi: 10.1038/s41598-021-93422-2 (PMC8260778; doi:10.1038/s41598-021-93422-2)
Supplement: Supplementary file 1 — Supplementary Information 1. [file 41598_2021_93422_MOESM1_ESM.pdf]

Supplementary Information for Triggering avalanches by transverse perturbations in a rotating drum

Vicente Salinas[1], **Cristobal Quininao**[2], **Sebastián González**[3], **and Gustavo Castillo**[2,*]

[1]Instituto de Ciencias Químicas Aplicadas, Inorganic Chemistry and Molecular Material Center, Facultad de Ingeniería, Universidad Autónoma de Chile, Santiago, Chile
[2]Instituto de Ciencias de la Ingeniería, Universidad de O'Higgins, Rancagua, Chile
[3]Dipartimento di Elettronica Informazione e Bioingegneria, Politecnico di Milano, Milan, Italy
[*]gustavo.castillo@uoh.cl

ABSTRACT

Supplementary information studying the role of the contact network and the potential energy on explaining the transition between the continuous and avalanching regime for the slowest rotating system we studied.

Contact network and elastic-gravitational energy

To study the effect of large amplitude A at small rotational speed we focus on the contact network structure, and a proxy for it, the elastic energy. Studying the contact network as a function of time is quite expensive computationally, and not too informative since we are interested in the bulk behavior and not in the spatial structure of the contact network. It is also highly correlated with the elastic energy ($R^2 \sim 0.7$). This can be understood since the distribution of overlaps is not very wide. Most of the contacts have an overlap very similar to the mean overlap, see Fig. 1.

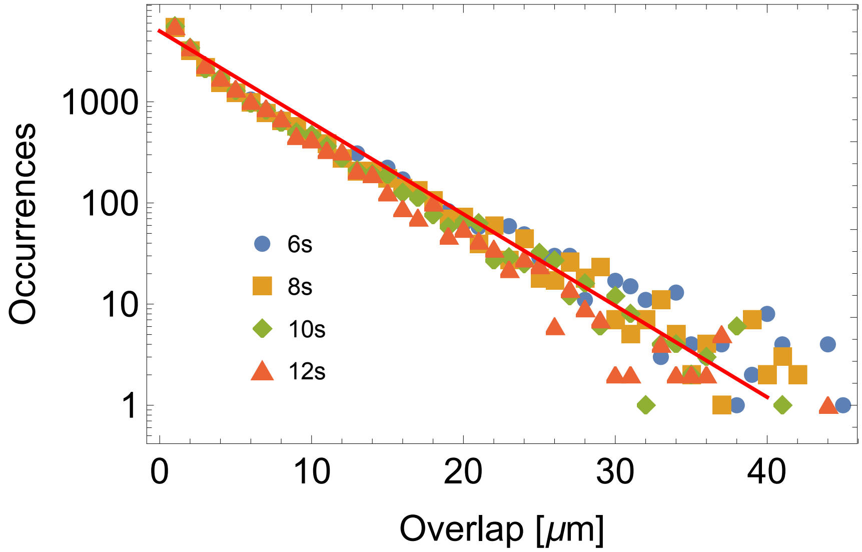

Figure 1. Overlap distribution in the system for a forcing with $A = 0.277\,\mu\text{m}$ at $120\,\text{Hz}$ for four different times. The red line is an exponential fit to the data. The mean of the overlap is $4.5\,\mu\text{m}$.

Figure 2 (left) shows the evolution of the number of contacts as a function of time for two simulations with the slowest rotating speed (1 rpm) and different forcing amplitude. This is highly correlated with the mean of the overlap squared, right panel. Since the latter quantity is proportional to the elastic energy of the system we use this quantity as an indicator of the state of the contact network. This simplifies the analysis since there is just one value per time step compared to the $\mathcal{O}(n)$ values that come from the contact network at each time step.

Despite correlating highly with the contact number, the elastic energy of the system is rather noisy. This is because the

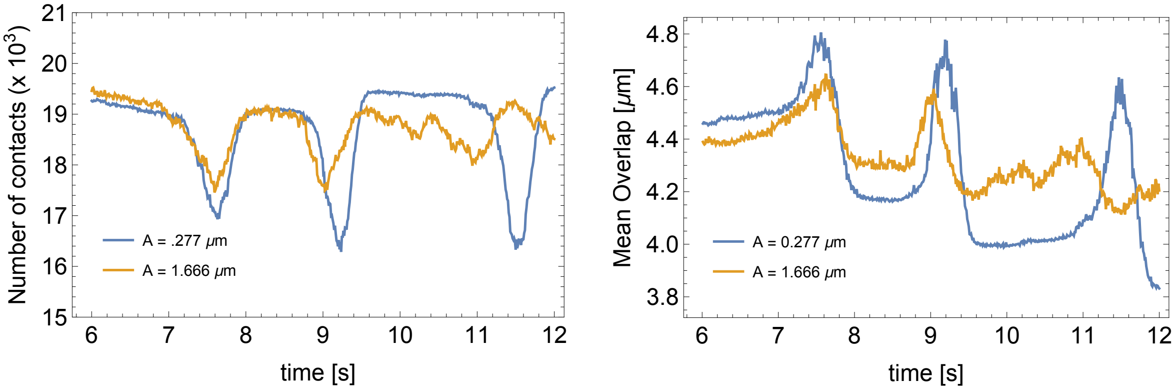

Figure 2. Left, total number of contacts in the system for two different forcing amplitudes, $A = 0.277\,\mu\text{m}$ and $A = 1.666\,\mu\text{m}$. Right, average of the overlap of all the contacts for the same systems. The correlation between the two signals is quite high for each system, therefore the elastic energy can be used as a proxy for the number of contacts.

contacts fluctuate in a time scale comparable to t_c. However, this variation is macroscopically insignificant.

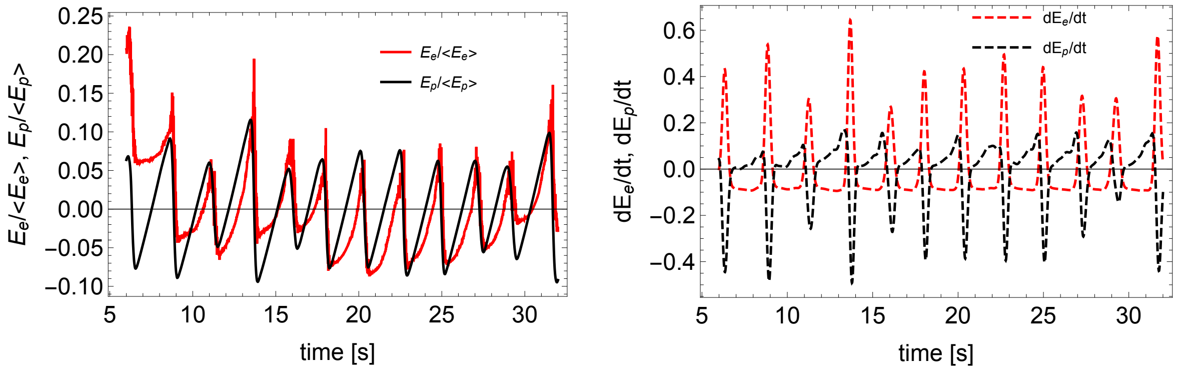

Figure 3. Evolution of the elastic E_e and potential E_p energies (normalised by their mean value) as a function of time for the system with forcing of $A = 0.277\,\mu\text{m}$, right panel. On the left, the time derivative of the smoothed signal.

To simplify even more the analysis we compute the potential energy of the system. We expect that the decrease of the potential energy will be correlated with the sudden change in the contact network of the system. Since the gravitational energy varies in the order of seconds (the time it takes a particle to move its diameter under gravity), the signal is much cleaner than for the elastic energy so we study the behaviour of the former in what follows. And rather than studying the value of the energies directly, we study their derivatives. This because the absolute value from avalanche to avalanche can vary but the slope at which the potential energy is lost or gained remains constant. In other words, there are avalanches that are shorter or longer than the average but the mechanism of these avalanches is always the same. This is also the reason why we don't study the Fourier transform of the signal and rather we focus on the distributions of values of the derivatives. Despite the fact that the evolution of the potential and elastic energy look the same, on would expect that the elastic energy changes first and is followed by the potential energy, indicating that the contact network is broken and triggers the avalanche. To confirm this we compute the lagged correlation between the elastic and the potential derivatives, Fig. 4. The evolution of the potential energy is indeed delayed with respect to the elastic energy by 8 time steps, confirming our hypothesis.

Fig. 5 shows the histograms of the variation of the potential energy for different forcing amplitudes at the same rotational speed and forcing frequency (120 Hz). The figure shows two distinct regimes, although the transition between them seems rather continuous. On the one hand the regime of continuous avalanche is characterised by a Gaussian distribution of the change in the potential energy. This can be seen clearly for the largest amplitude of the set, $6.66\,\mu\text{m}$. As the avalanches become discontinuous the distribution of the change in the potential energy becomes asymmetrical: the system spends most of the time moving as a rigid solid, and therefore dE_P/dT is positive, as can be seen from the distributions with a peak on the right of the plot. Since in the discontinuous regime the particles move like a rigid solid they will all gain potential energy at the same rate. This makes that for small amplitude all the system have the same peak in the distribution of potential energy variation. As the forcing amplitude increases the avalanches are triggered at a lower angle thus shifting the peak eventually towards zero, as can

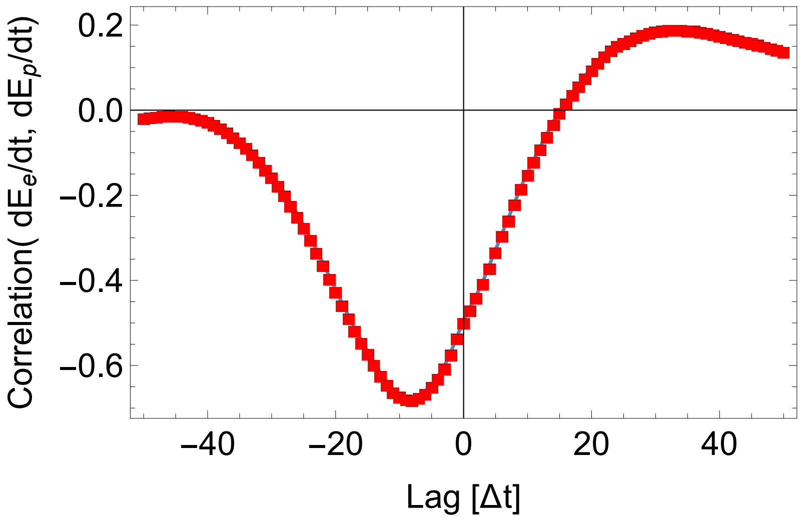

Figure 4. Delayed correlation between the derivative of the elastic and the potential energy (Fig. 3 left). The minimum of the the correlation is at $\Delta t = 8$, meaning that the potential energy is delayed with respect to the elastic energy by 0.16s.

be seen in the inset of Fig. 5.

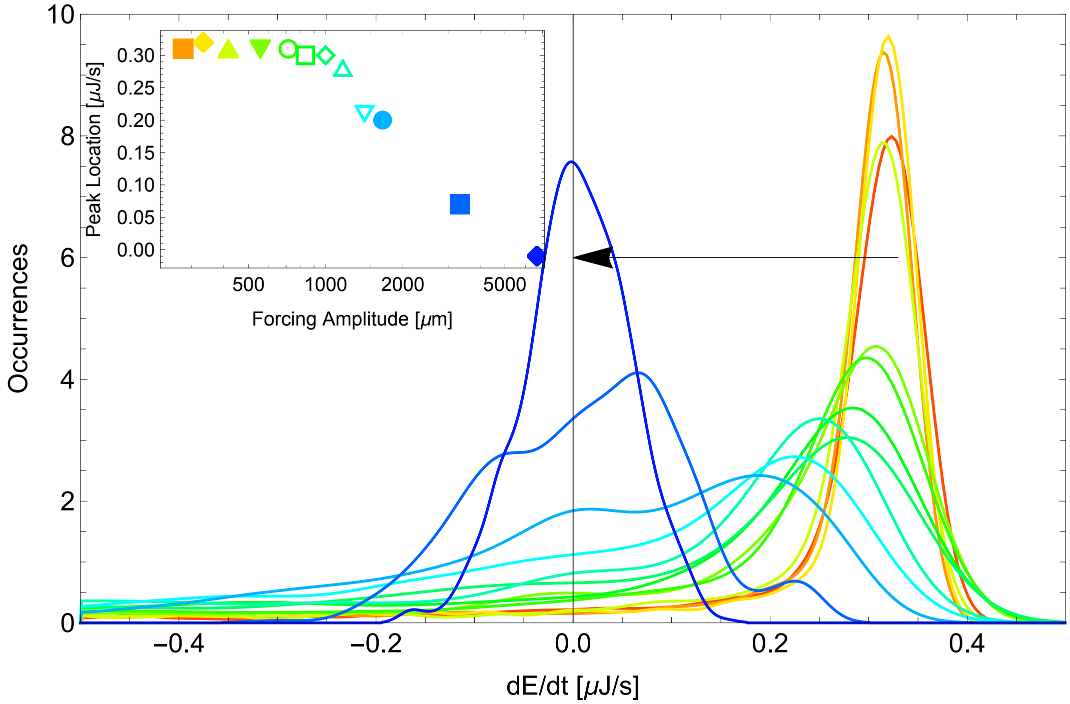

Figure 5. Smoothed histograms for the variation of potential energy dE_p/dt between 6s and 32s increasing the amplitude of the forcing at constant frequency of 120Hz, from 0 (red color) to 6.66 μm. On the inset we show how the peak location moves as the forcing amplitude increases. The colors in the histogram correspond to the values shown on the inset.

Comparison between the kinetic energy and the function $v(t)$

As can be seen from Fig. 6, the behavior of the function $v(t)$ displays the same qualitative features as $K(t)$. It shows the peaks well as the fast increment/slow drops, just what we observe in the kinetic energy.

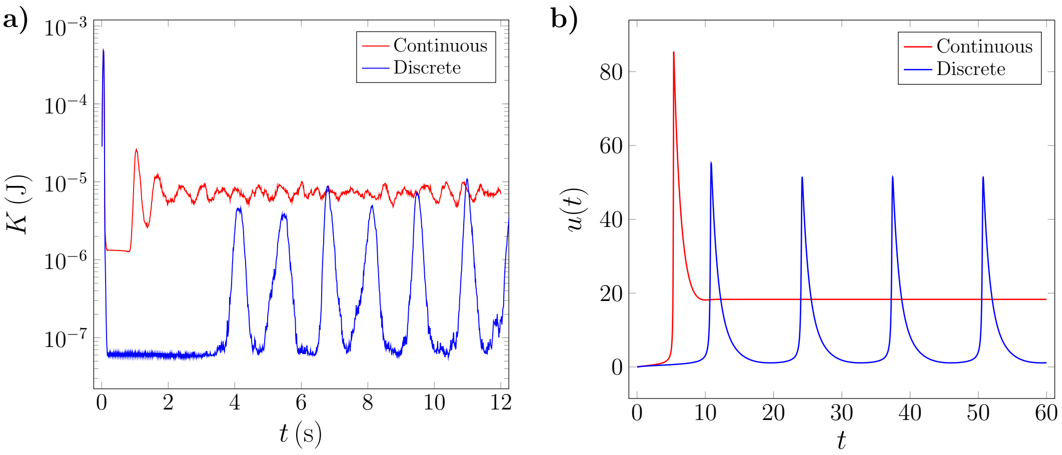

Figure 6. a) Evolution of the kinetic energy K for $A = 0.8\,\mu$m and $\Omega = 2$ rpm and $\Omega = 10$ rpm for the discrete and continuous cases respectively. **b)** Evolution of the function $v(t)$ of the theoretical model.

Bifurcation analysis

For the proposed model:

$$\begin{aligned} \dot{v} &= f(A) - v + u + v^2 u, \\ \dot{u} &= \varepsilon\left(g(\Omega) - u - v^2 u\right), \end{aligned}$$

the equilibrium of the system is

$$v^* = f(A) + g(\Omega), \quad u^* = \frac{g(\Omega)}{1 + (f(A) + g(\Omega))^2}.$$

If we change coordinates by letting $x = v - f(A) - g(\Omega)$, and $y = u - \frac{g(\Omega)}{1+(f(A)+g(\Omega))^2}$, then $v = x + v^*$ and $u = y + u^*$, the system becomes:

$$\begin{aligned} \dot{x} &= f(A) - (x + f(A) + g(\Omega)) + \left(y + \frac{g(\Omega)}{1 + (f(A)+g(\Omega))^2}\right) + (x + f(A) + g(\Omega))^2 \left(y + \frac{g(\Omega)}{1+(f(A)+g(\Omega))^2}\right), \\ \dot{y} &= \varepsilon\left(g(\Omega) - \left(y + \frac{g(\Omega)}{1+(f(A)+g(\Omega))^2}\right) - (x + f(A) + g(\Omega))^2 \left(y + \frac{g(\Omega)}{1+(f(A)+g(\Omega))^2}\right)\right), \end{aligned}$$

and the equilibrium is now $(x^*, y^*) = (0,0)$. The Jacobian matrix at the equilibrium is

$$\left.\begin{pmatrix} -1 + 2(x + f(A) + g(\Omega))\left(y + \frac{g(\Omega)}{1+(f(A)+g(\Omega))^2}\right) & 1 + (x + f(A) + g(\Omega))^2 \\ -2\varepsilon(x + f(A) + g(\Omega))\left(y + \frac{g(\Omega)}{1+(f(A)+g(\Omega))^2}\right) & -\varepsilon - \varepsilon(x + f(A) + g(\Omega))^2 \end{pmatrix}\right|_{(x,y)=(0,0)} =$$

$$\begin{pmatrix} -1 + 2(f(A) + g(\Omega))\left(\frac{g(\Omega)}{1+(f(A)+g(\Omega))^2}\right) & 1 + (f(A) + g(\Omega))^2 \\ -2\varepsilon(f(A) + g(\Omega))\left(\frac{g(\Omega)}{1+(f(A)+g(\Omega))^2}\right) & -\varepsilon - \varepsilon(f(A) + g(\Omega))^2 \end{pmatrix}$$

To compute the eigenvalues, we use the trace determinant plane. The determinant of the Jacobian at the equilibrium is

$$\det(J) = \varepsilon(1 + (f(A) + g(\Omega))^2) > 0,$$

and the trace

$$\operatorname{tr}(J) = -\frac{1}{1 + (f(A) + g(\Omega))^2}\left(1 + (f(A) + g(\Omega))^2 - 2g(\Omega)(f(A) + g(\Omega)) + \varepsilon(1 + (f(A) + g(\Omega))^2)^2\right).$$

If $f(A)=0$, the sign of the $\mathrm{tr}(J)$ is a result of

$$F(0,g(\Omega))=\varepsilon g(\Omega)^4+(2\varepsilon-1)g(\Omega)^2+1+\varepsilon$$

taking the derivative for finding the minima leads to

$$4\varepsilon g(\Omega)^3+2(2\varepsilon-1)g(\Omega)=0,$$

with solutions

$$g(\Omega)_0=0,\quad g(\Omega)_\pm=\pm\sqrt{\frac{1-2\varepsilon}{2\varepsilon}},$$

getting the first condition $\varepsilon<1/2$. However, that condition is not sufficient because to have a well defined $\mathrm{tr}(J)=0$ curve, a necessary conditions is that the minimum $F(f(\Omega_+))<0$:

$$-\frac{(1-2\varepsilon)^2}{4\varepsilon}+1+\varepsilon=\frac{-(1-2\varepsilon)^2+4\varepsilon(1+\varepsilon)}{4\varepsilon}=\frac{-1+8\varepsilon}{4\varepsilon}<0\quad\Rightarrow\quad\varepsilon<\frac{1}{8}.$$

On the other hand, notice that

$$F(f(A),g(\Omega))=1+(f(A)+g(\Omega))^2-2g(\Omega)(f(A)+g(\Omega))+\varepsilon(1+(f(A)+g(\Omega))^2)^2,$$

is such that

$$\partial_{f(A)}F(f(A),g(\Omega))=2f(A)+4\varepsilon(f(A)+g(\Omega))(1+(f(A)+g(\Omega))^2)>0$$

is increasing in $f(A)$, thus the condition $\varepsilon<\frac{1}{8}$ is implying that the equation $\mathrm{tr}(J)=0$ defines a regular curve on the $(f(A),g(\Omega))$. Finally, the eigenvalues of the Jacobian matrix are

$$\lambda_\pm=\alpha(f(A),g(\Omega))\pm\beta(f(A),g(\Omega)),\quad\text{with}\quad\alpha(f(A),g(\Omega))=\frac{\mathrm{tr}(J)}{2},\quad\beta=\frac{1}{2}\sqrt{\mathrm{tr}(J)^2-4\det(J)}.$$

Whenever $\mathrm{tr}(J)=0$ - which itself defines the bifurcation curve of Figure 3.b) main text - we obtain a pair of pure imaginary eigenvalues, corresponding to the non-hyperbolicity condition of the Hopf Bifurcation Theorem. All in all, for ε small, the system fulfills the Hopf Bifurcation Theorem conditions, and the self-oscillation analogy indeed present a Hopf bifurcation whenever Ω or A are large enough.
